# A code with a twist: supraparticle microrod composites with direction dependent optical properties as anti-counterfeit labels†

**DOI:** 10.1039/c8na00334c

**Published:** 2019-01-31

**Authors:** Susanne Wintzheimer, Tim Granath, Antonia Eppinger, Manuel Rodrigues Goncalves, Karl Mandel

**Affiliations:** Chair of Chemical Technology of Materials Synthesis, Julius-Maximilians-University Würzburg Röntgenring 11 D97070 Würzburg Germany; Institute of Experimental Physics, Ulm University Albert-Einstein-Allee 11 D-89069 Ulm Germany; Fraunhofer Institute for Silicate Research, ISC Neunerplatz 2 D97082 Würzburg Germany karl-sebastian.mandel@isc.fraunhofer.de

## Abstract

Superparamagnetic iron oxide nanoparticles can be assembled to form anisotropic microrod supraparticles with the assistance of a magnetic field during synthesis. Optionally, these iron oxide microrods can furthermore be coated with a thin silica shell. Due to their anisotropic structure, both microrod types can be aligned in a magnetic field while being dispersed in a matrix material which can be cured during the alignment of the microrods. In this way, an anisotropic magnetic composite is obtained. Interestingly, it was observed that the optical extinction properties for visible light in such a composite are direction dependent, which can be explained by using appropriate models based on Maxwell equations. Based on the understanding of this principle, a clever approach for a hidden code could be proposed which is obtained from mixing pure iron oxide and silica coated microrod supraparticles in such an anisotropic composite. The hidden code, which comes down to obtaining a single value eventually, can only be revealed when knowing that the system needs to be measured with a certain “twist”.

## Abstract 2

Superparamagnetic microrod supraparticles can be aligned in a magnetic field whose direction can be fixed in a matrix. These anisotropic composites reveal anisotropic optical properties, usefully to create anti-counterfeit labels.

## Graphical abstract

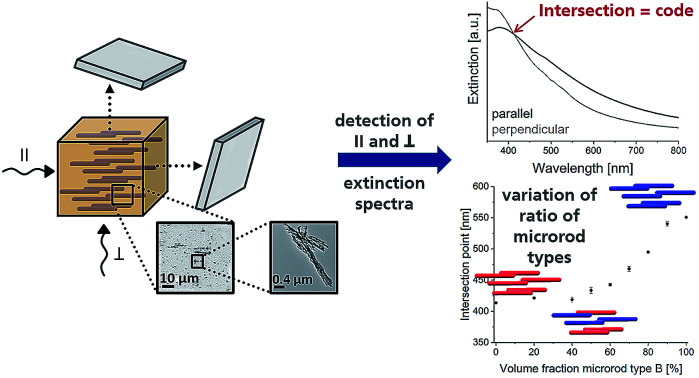

## Introduction

Counterfeiting causes severe trouble for society as it not only violates the trade mark rights of the owner but also threatens the health of patients in the case of counterfeited drugs and damages the economy in the case of counterfeited banknotes and other high-value products, for instance.^1–3^ Therefore, a vast variety of security labels have been developed including watermarks, holograms, fine prints and security inks.^1,4,5^ However, due to the advances in technology, most labels are already easy to copy for counterfeiters.^3^ This is why new materials and methods for anti-counterfeiting have to be developed which preferably bear codes invisible to the public, only readable by a smart analytical system.^4,6^ Even more ideally, an object that contains a code should be designed in a way that the information can only be obtained when a read-out procedure is done in the right way, *i.e.*, the object is designed in a way that it is rather unspectacular in appearance but conceal a secret code that is only revealed upon “asking the right question”. There are basically two ways of coding: either “graphically” or “optically”.^7,8^ In this context, “graphically” means that a code is obtained from spatially distributing certain marks in an object, *i.e.*, it uses the principle of a barcode. “Optically” means that the integral information obtained from an object is collected at once and then, by using an appropriate detector, this signal is deconvoluted in a spectrum which contains certain characteristics (for instance certain fluorescence peaks in the visible spectrum, *i.e.*, a series of distinct colours).^7,8^ Nano- and nanostructured objects have been developed for both approaches and recent reviews give an excellent overview on the most important developments in this field.^7,9^ Besides being sophisticated, the demand from applicants, however, is that the labels are cheap and simple and also the read-out procedure must not be too complex, expensive or time consuming.^5,10^

Herein, we present a system which is made in a very simple way and which breaks with the classical distinction between the two approaches “graphical” and “optical” codes and mixes both principles. For this purpose, a mixture of two nanostructured objects is spatially distributed in a matrix (=“graphical code” principle). Then, the *integral* signal needs to be read out (=“optical code” principle), however, very importantly, from two directions (=“digital graphical code” principle). This ultimately results in one single value obtained with the detector (=neither graphical nor optical but simply a single number result). This single value eventually depends on the ratio of the initially spatially distributed two objects and therefore can be adjusted in a way that a code can be maintained by selecting a combination of the two objects.

## Experimental

### Materials

Iron(iii)chloride hexahydrate (FeCl_3_·6H_2_O, 99%), iron(ii) chloride tetrahydrate (FeCl_2_·4H_2_O, 99%) and ammonia solution (aqueous NH_3_, 28–30 wt%) were obtained from Sigma-Aldrich, Germany. Cyclohexane (100%) was purchased from VWR International, Germany and tetraethylorthosilicate (TEOS, 99%) from abcr GmbH, Germany. Nitric acid (HNO_3_, 65% solution) was ordered from Otto Fischar GmbH & Co. KG, Germany and agar agar was obtained from Biovegan GmbH, Germany. All reagents were used without further purification. Magnetic alignment experiments were carried out with a neodymium permanent magnet (Q-51-51-25-N) obtained from http://www.supermagnete.de.

### Microrod synthesis and their matrix incorporation

Superparamagnetic microrods (type A) were obtained by a two-step process as already described elsewhere.^11^ First, superparamagnetic iron oxide nanoparticles were synthesised *via* the co-precipitation method. In brief, 8 mmol FeCl_3_·6H_2_O and 4 mmol FeCl_2_·4H_2_O were dissolved in 100 mL deionized water. After a quick addition of 5 mL aqueous ammonia, the immediately formed black precipitate was magnetically separated, decanted, and washed until neutral pH. The obtained nanoparticles were redispersed by addition of 10 mL nitric acid (1 M) and further diluted with 100 mL water. In the second step, 1 mL TEOS was added dropwise into 20 mL of the as-prepared ferrofluid and slowly overlaid with 10 mL cyclohexane. Then, 125 mL acetone was quickly poured into the mixture while the whole system was exposed to a magnetic field. Finally, the obtained precipitate was magnetically separated, decanted and washed once with 2.5 mL NaOH (0.01 M) in 75 mL acetone, and then twice with an acetone–water-mixture (2 : 1) and twice with H_2_O. Redispersion was performed with 100 mL H_2_O acidified with 40 μL nitric acid (1 M) for further stabilization.

To obtain silica-coated superparamagnetic microrods (type B), 40 mL ethanol and 1 mL ammonia were added to 10 mL of the as-prepared type A microrod dispersion. Then, 100 μL TEOS was added dropwise into the reaction mixture. This recipe is a modified version of the well-known Stöber synthesis to create colloidal silica. However, due to the selected very low concentrations of TEOS, the condensation of the hydrolysed ethxoy-silane is of the heterogenous type, *i.e.*, no silica nanoparticles are formed homogenously in solution but rather a heterogenous Si-O_*x*_ nucleation takes place on the microrod surface, leading to the growth of a thin silica shell on the surface of the iron oxide. After 8 h agitation, the product was magnetically separated, decanted and washed with H_2_O and finally redispersed in 10 mL H_2_O.

In order to incorporate the synthesised microrods into a matrix, the obtained supraparticle dispersion (type A or B) was diluted to a volume of 200 μL with a pH-adjusted HNO_3_-solution (pH = 3.6) and 3.5 g of a hot agar solution (25 mg agar dissolved in 5 mL H_2_O) was added and intermixed within a cuvette. The cuvette was closed and cured at room temperature standing next to a magnet at a distance of 13 cm. To investigate the anisotropic optical properties, UV/Vis measurements were performed, having the microrods orientated once parallel and once perpendicular to the beam path, simply by rotating the cuvette by 90°.

### Characterization

UV/Vis analysis was performed with a Specord 50 spectrometer from Analytik Jena using polystyrene cuvettes with four transparent sides. The magnetic properties of the superparamagnetic microrods were studied with a vibrating sample magnetometer (VSM, VersaLab 3 T Cryogen-free vibrating sample magnetometer) by cycling the applied field from −30 to +30 kOe two times with a step rate of 100 Oe s^−1^. The temperature was set to 20 °C. Optical images were obtained using a Keyence 3D Laser Scanning Confocal Microscope VK-X 200 series (controller: VK-X 200, measuring unit: VK-X 210). TEM images were obtained on a JEOL JSM 2010 at an acceleration voltage of 200 kV (with samples prepared on carbon-coated copper grids).

## Results and discussion

**Fig. 1:**
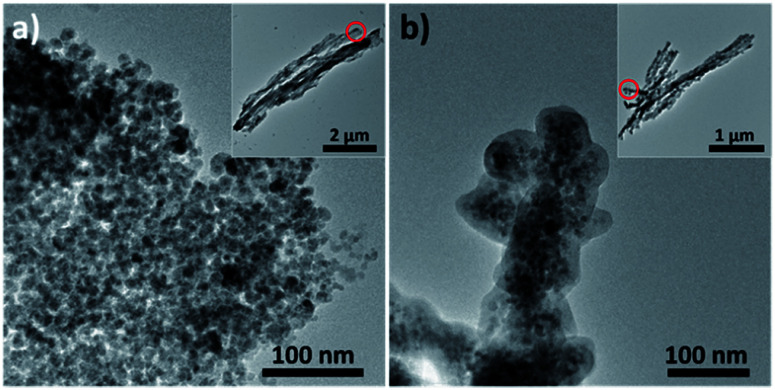
TEM images of (a) iron oxide microrod supraparticles (type A) and (b) microrod supraparticles with a silica shell (type B). Insets show the overview of several microrods with red circles indicating the areas that are depicted with a higher magnification in the large images.

**Fig. 2:**
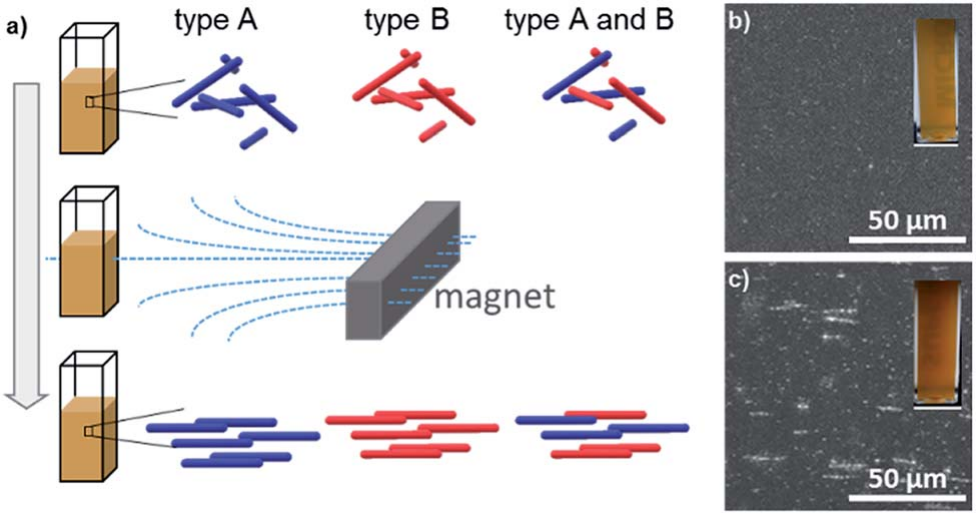
(a) Scheme of the alignment of microrods in a matrix to obtain an object with optical anisotropy and (b), (c) LSM images and photographs (insets) of magnetically aligned type A microrod–matrix composites (viewing direction (b) parallel and (c) perpendicular to the microrods).

The nanostructured objects used to create the composite code object are superparamagnetic microrod supraparticles. Supraparticles in general specify hierarchically structured particles formed from prefabricated nanoparticles. This structural arrangement generates additional functionalities, which the plain nanoparticles have not possessed before.^12–15^ In our specific case, the obtained supraparticles possess an anisotropic microrod-like shape and thus yield magnetic alignability while still being superparamagnetic. The synthesis of the basic type A microrod has been published earlier:^11,16^ the procedure is simply to add an anti-solvent and traces of silica to an aqueous ferrofluid (a sol of about 10 nm sized superparamagnetic iron oxide nanoparticles) in the presence of an external magnetic field. These microrods of type A (Fig. 1a) contain traces of silica which glue the iron oxide nanoparticles together; however, as >90% of the microrods consist of iron oxide nanoparticles, they can be considered in a first approximation to be composed of pure iron oxide. The approximate dimensions of type A microrods are 20 to 100 nm in diameter and 100 nm to 10 μm in length. However, it is also possible to enclose the microrod supraparticles of type A fully in a dense, about 10 nm thick shell of amorphous silica (which increases each dimension of a microrod by 20 nm). To achieve this, an appropriate synthesis procedure was developed in this current work (see the Experimental section for details). The silica-shelled microrods are of type B (Fig. 1b). It is worth noting that the magnetic properties of the type B microrods slightly decreased as expected when adding a non-magnetic material (silica) to the system (for a comparison of the magnetisation measurements of the two superparamagnetic systems, see Fig. S1 in the ESI†). However, both microrod types (A and B) are sufficiently magnetic to be easily manipulable in dispersion by using a standard handheld magnet. Being magnetically directable in an external magnetic field is the most crucial precondition to be able to create a composite, ultimately being useful as a code-object, as will be described in the following: by placing a magnet next to a dispersion of the microrod supraparticles (either of type A or B or a quantitative mixture of both) and then solidifying the matrix in which the microrods are dispersed (see the Experimental section), the position of the microrods, which are aligned parallel to the field lines of the external magnetic field applied, can be permanently fixed (see the scheme in Fig. 2a). The success of the magnetic field induced and subsequently fixed alignment of the microrods could be confirmed by laser scanning microscopy (Fig. 2b and c) while the composites visually simply have a brownish appearance (insets Fig. 2b and c).

**Fig. 3:**
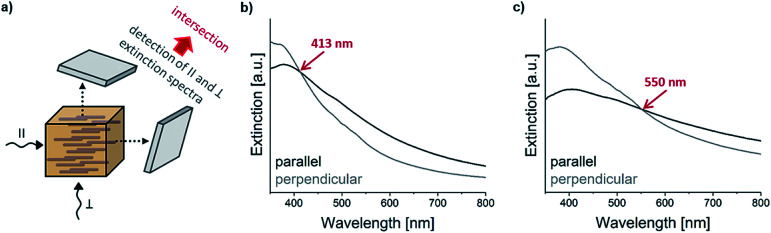
(a) Scheme showing the two directions of the light beam (parallel ‖ and perpendicular ⊥ to the microrods) during extinction measurements as well as ‖ and ⊥ extinction curves of samples containing (b) type A (iron oxide) and (c) type B (silica-coated iron oxide) microrods in a matrix.

When studying the extinction spectra of the composites, containing microrods of either only type A or only type B, it was found that they differ, depending on whether the microrods are parallel or perpendicularly aligned in the composite with respect to the light propagation (see Fig. 3a for the measurement setup). As the microrods are anisotropic, an anisotropic behaviour of the composite was expected. However, surprisingly, the extinction was not shifted equally over all wavelengths but rather non-linearly. Thus, from superimposing the recorded extinction spectra measured in parallel and perpendicular directions relative to the microrod alignment, an intersection in the spectral curves is obtained. Interestingly, the position of this intersection is at considerably lower wavelengths for type A microrods (Fig. 3b), namely at 413 nm, compared to type B microrods (Fig. 3c) which is at 550 nm. It is worth noting that the extinction measurements do not necessarily need to be exactly parallel or perpendicular with respect to the microrod direction; in fact, the intersection is invariant (*i.e.*, always is at the same wavelength) for any two superimposed extinction spectral curves obtained from having measured the composite from any two angles. Only the width of splitting of the intensity of the curves below and above the wavelength of intersection varies as a function of the angle between the two directions of measurement (Fig. S2 in the ESI†). It should also be noted that the signal can be obtained in a well reproducible way (at least 5 measurements were carried out from which a variance below 10 nm in wavelength was obtained): it is insensitive to slight changes during preparation and easy to measure, but with the special feature of an unexpected signal behaviour.

**Fig. 4:**
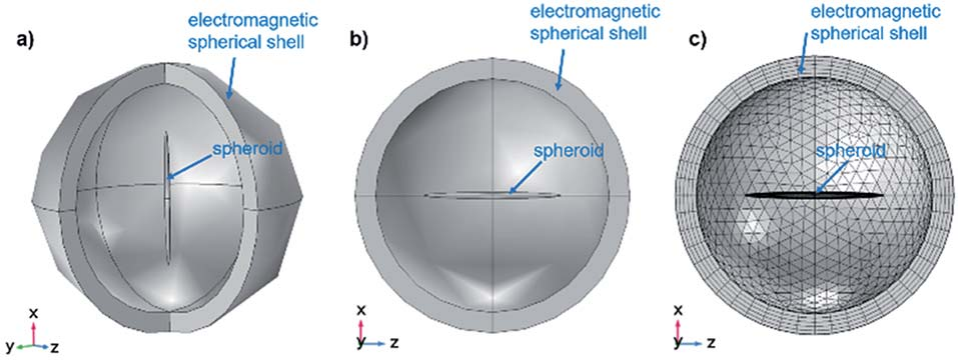
(a) 3D representation with two quadrants dropped for better understanding and (b) cross section in the *xz* plane of the model of the geometry for computation, consisting of one spheroid inside a spherical domain of the homogeneous refractive index used for the simulations of the light absorption and scattering by the microrods with COMSOL. (c) Cross section in the *xz* plane of the geometry showing the meshing used to solve the scattering problem.

In order to understand this complex behaviour of light interaction with the microrods, theoretical modelling was performed based on full electromagnetic calculations (Maxwell equations) using the computer program COMSOL Multiphysics. For theoretical modelling, the following parameters were chosen: The microrod is represented by a homogeneous spheroid with a diameter of 100 nm and a length of 2 μm. For simplification the surrounding medium is assumed to be water (refractive index *n* = 1.33) instead of agar-agar. The calculations used a spherical computational domain of diameter *D* = 4 μm. A perfectly matching layer (PML) of 300 nm thickness was used to attenuate the scattered light from the spheroids at the external boundary. The outer boundary was defined as a scattering boundary condition, adequate for solving the scattering problems. The excitation used is a plane wave of electric field amplitude 1 V m^−1^. Graphical representations of the computational domain used in all calculations are shown in Fig. 4.

The spheroid has its longest axis parallel to the *x*-axis, or along the *z*-axis. The direction of the excitation plane wave (*k*-vector) is along the negative *z*-axis. The polarization direction was chosen, either along the *x*-axis (parallel to the spheroid), or along the *y*-axis (perpendicular to the spheroid), or at 45° to the *x*-axis. The materials used in the simulations were Fe_2_O_3_ or Fe_3_O_4_ to take the different oxidation states of Fe(iii) and Fe(ii) of iron oxide into account, potentially present in the synthesised microrods. The optical constants (*n* – real value and *k* – imaginary value) of these materials, required for solving the electromagnetic problem, were taken from the publication of M. R. Query,^17^ which were made available online at https://refractiveindex.info/. As Fe_2_O_3_ is a birefringent material, simulations were carried out with *n* and *k* both for ordinary and extraordinary rays.

**Fig. 5:**
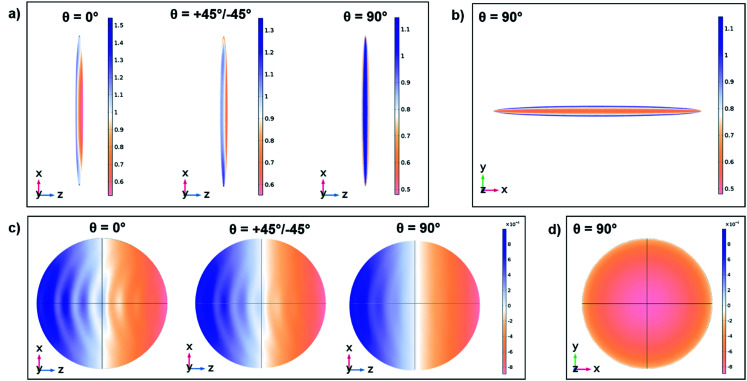
COMSOL simulated near-fields at the surface of the Fe_3_O_4_ spheroid for the *k*-vector of the plane wave with a wavelength of 600 nm (a) perpendicular or (b) parallel to the spheroid and the time averaged power outflow crossing for the *k*-vector of the plane wave (c) perpendicular or (d) parallel to the spheroid. For both angles of light propagation, the near-fields and power outflow crossings for the 4 polarization angles *θ* = −45°, 0°, 45°, 90° are depicted.

COMSOL permits to obtain the near-fields inside, at the surface and surrounding the spheroids, and the far-fields at the internal boundary of the PML layer in order to calculate the total cross sections (scattering, absorption and extinction). The distributions of the electric near-field at the surface of the spheroids (exemplarily for the case of Fe_3_O_4_) for an excitation wavelength of 600 nm are presented in Fig. 5a and b. In Fig. 5c and d the power outflow (time averaged Poynting vector) at the inner surface of the PML spherical surface is shown (exemplarily for the case of Fe_3_O_4_). For this calculation, COMSOL takes a spherical imaginary surface at a very long distance compared to the wavelength and size of the scattering spheroid. The blue colour represents far-field light leaving the boundary, whereas the yellow colour corresponds to light entering the spherical boundary (Fig. 5c), in agreement with the definition of the plane wave propagation direction. The surface patterns obtained for incident light polarized parallel or perpendicular to the spheroid show considerable differences in the distribution of fringes. This is due to the scattering properties of the elongated spheroid interacting with light at two different polarizations. These patterns are presented for qualitative comparison only. The quantitative experimental characterization of the radiation patterns for a well-defined polarization direction of the excitation would require an angle resolved spectroscopy technique.

**Fig. 6:**
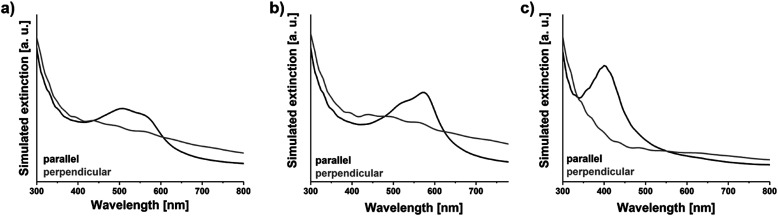
COMSOL simulated extinction cross sections for Fe_2_O_3_ for (a) extraordinary and (b) ordinary rays and (c) Fe_3_O_4_ in the case of parallel and perpendicular light propagation with respect to the spheroids.

As the simulations were done with polarized light for the illumination of the spheroid, the extinction cross sections are also dependent on the polarization direction. In order to achieve a better comparison with experimental results, where non-polarized light was used, the cross sections were averaged over 4 different polarization angles (−45°, 0°, 45°, and 90°, see Fig. S3 in the ESI†). The obtained simulated extinction cross sections for Fe_2_O_3_ (ordinary and extraordinary rays) and Fe_3_O_4_ in the case of parallel and perpendicular light propagation with respect to the orientation of the spheroids are depicted in Fig. 6. All simulated cross sections show an extinction of light by the microrods in the complete studied wavelength range (300 to 800 nm) while the extinction intensity depends on the direction and wavelength of the propagating light as well as on the spheroid material. The simulated extinction accounts for the absorption and scattering of the studied materials (which is exemplarily shown for a Fe_3_O_4_ spheroid in Fig. S4 in the ESI†). The acquired graphs underline the anisotropic extinction behaviour of the studied spheroid with a higher extinction at shorter wavelengths (∼300 to 450 nm) of light propagating perpendicular in comparison to light parallel. This behaviour switches at longer wavelengths (∼450 to 600 nm), resulting in an intersection of both curves (at ∼350 or 450 nm depending on the material), which correlates with the obtained experimental outcomes (Fig. 3). However, a second intersection at longer wavelength ranges (at ∼550 or 600 nm depending on the material) of the simulated extinction cross sections does not reflect the experimental findings.

This discrepancy may on the one hand be explained by the shift of the curves one notices when comparing the theoretical with the experimental extinction curves: the experimentally assessed intersection is found at longer wavelengths indicating that a second intersection may also exist but has not been measured as it is out of the range of the measurement system at over 800 nm. On the other hand, in the simulated examples only one geometry was calculated. Changes in the extinction cross-sectional spectra for smaller or larger spheroids or for another refractive index of the surrounding medium are possible. The simulations do not consider the polydispersity of the particle size either. Nevertheless, the theoretical modelling is supporting the experimental results as it clearly shows an anisotropic extinction behaviour of the microrods due to their shape, resulting in at least one intersection of the extinction as a function of the wavelength of light propagating parallel and perpendicular to the microrods. Additionally, it underlines the material dependence of the extinction spectra and therefore of the position of the intersection.

**Fig. 7:**
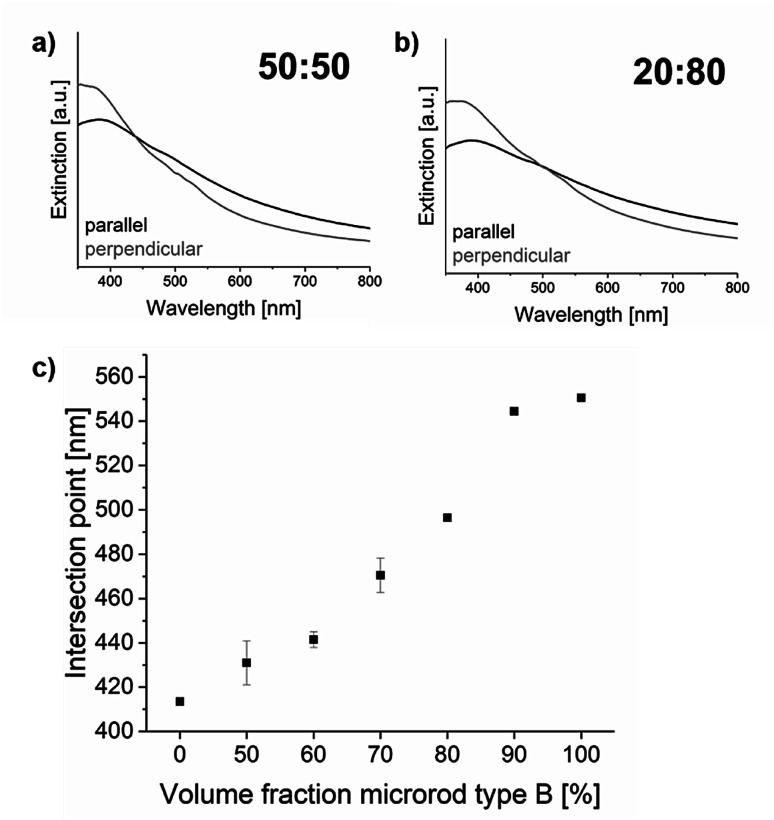
The ‖ and ⊥ extinction curves of samples containing mixtures of type A (iron oxide) and B (silica-coated iron oxide) microrods in different ratios A : B (v%/v%) in a matrix: (a) 50 : 50 and (b) 20 : 80. (c) Depicts all measured intersections which were used for the creation of distinguishable codes dependent on the type A and B ratios.

Summing up so far, it has been found and – by means of theoretical modelling – verified (in terms of validity) that magnetically oriented microrods yield direction dependent extinction behaviour. This anisotropic behaviour can be exploited to determine a very well-defined point of intersection when measuring the spectral transmittance from two arbitrary but different directions. Moreover, the position of the intersection point is determined by the optical material properties of the microrods, which can be used to create unique codes. For this purpose, quantitative mixtures of microrods of type A and type B were prepared (namely: A : B (v%/v%) = 100 : 0, 50 : 50, 40 : 60, 30 : 70, 20 : 80, 10 : 90 and 0 : 100), aligned in a magnetic field and fixed in a matrix to obtain a solid composite. From this “graphical” code (=spacial arrangement of objects) an integral signal (transmitted light) was obtained and spectrally resolved (=“optical code”). However, only by performing this procedure at two different angles (=“binary graphically”; security feature that demands “asking the right question”), one unique intersection at one specific wavelength (=“numerical code”) could be obtained. The wavelength at which this intersection could be observed was found to correlate very well with the quantitative ratio of the mixtures of type A and type B microrods (Fig. 7). Thus, this ultimately allows predefining a unique code number by adjusting a certain mixture of the two microrod types, which can only be detected if the “reader” is aware of “the little twist”.

## Conclusions

In this work, magnetically assisted alignment of anisotropic superparamagnetic microrod supraparticles in a matrix yielded a nanocomposite material with direction dependent optical extinction properties, which can theoretically be explained using the principles of Maxwell equations in appropriate computer models. This finding could be exploited to create a unique code based on a mixture of silica coated and non-coated microrods in the composite, measured optically at two different angles. This first proof of principle shows that a handful of codes can thereby be generated. Modification of the microrod surface with chemical moieties other than silica might bring about further unexpected, direction dependent optical effects, potentially opening up a much wider framework of possibilities to create unique combinations of codes. Moreover, such optical effects might be of great interest for other optical applications where direction dependence is of importance, such as dichroic filters.

## Conflicts of interest

There are no conflicts to declare.

## Supplementary Material

NA-001-C8NA00334C-s001
