# Supplementary material for: A code with a twist: supraparticle microrod composites with direction dependent optical properties as anti-counterfeit labels
Source: Nanoscale Adv. 2019 Jan 31;1(4):1510–5. doi: 10.1039/c8na00334c (PMC9417921; doi:10.1039/c8na00334c)
Supplement: NA-001-C8NA00334C-s001 [file NA-001-C8NA00334C-s001.pdf]

Electronic Supplementary Material (ESI) for Nanoscale Advances.
This journal is © The Royal Society of Chemistry 2019

Supporting Information

A code with a twist: Supraparticle microrod composites with direction dependent optical properties as anti-counterfeit label

Susanne Wintzheimer,[a] Tim Granath,[a] Antonia Eppinger,[a] Manuel Rodrigues Goncalves,[b] and Karl Mandel*[,a,c]

a Chair of Chemical Technology of Materials Synthesis, Julius-Maximilians-University Würzburg, Röntgenring 11, D97070 Würzburg, Germany.
b Institute of Experimental Physics, Ulm University, Albert-Einstein-Allee 11, D-89069 Ulm, Germany.
c Fraunhofer Institute for Silicate Research, ISC, Neunerplatz 2, D97082 Würzburg, Germany. karl-sebastian.mandel@isc.fraunhofer.de

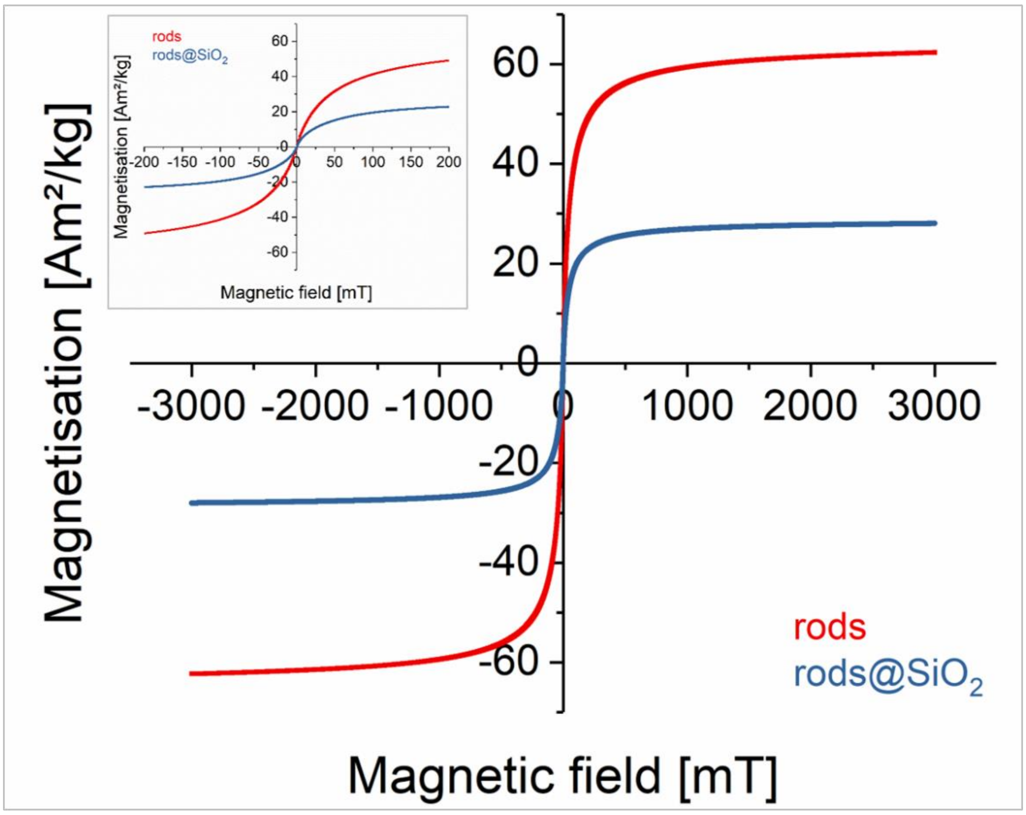

Figure S1. Magnetisation measurements of a) type A (iron oxide) and b) type B (iron oxide with a SiO_2 shell) microrods.

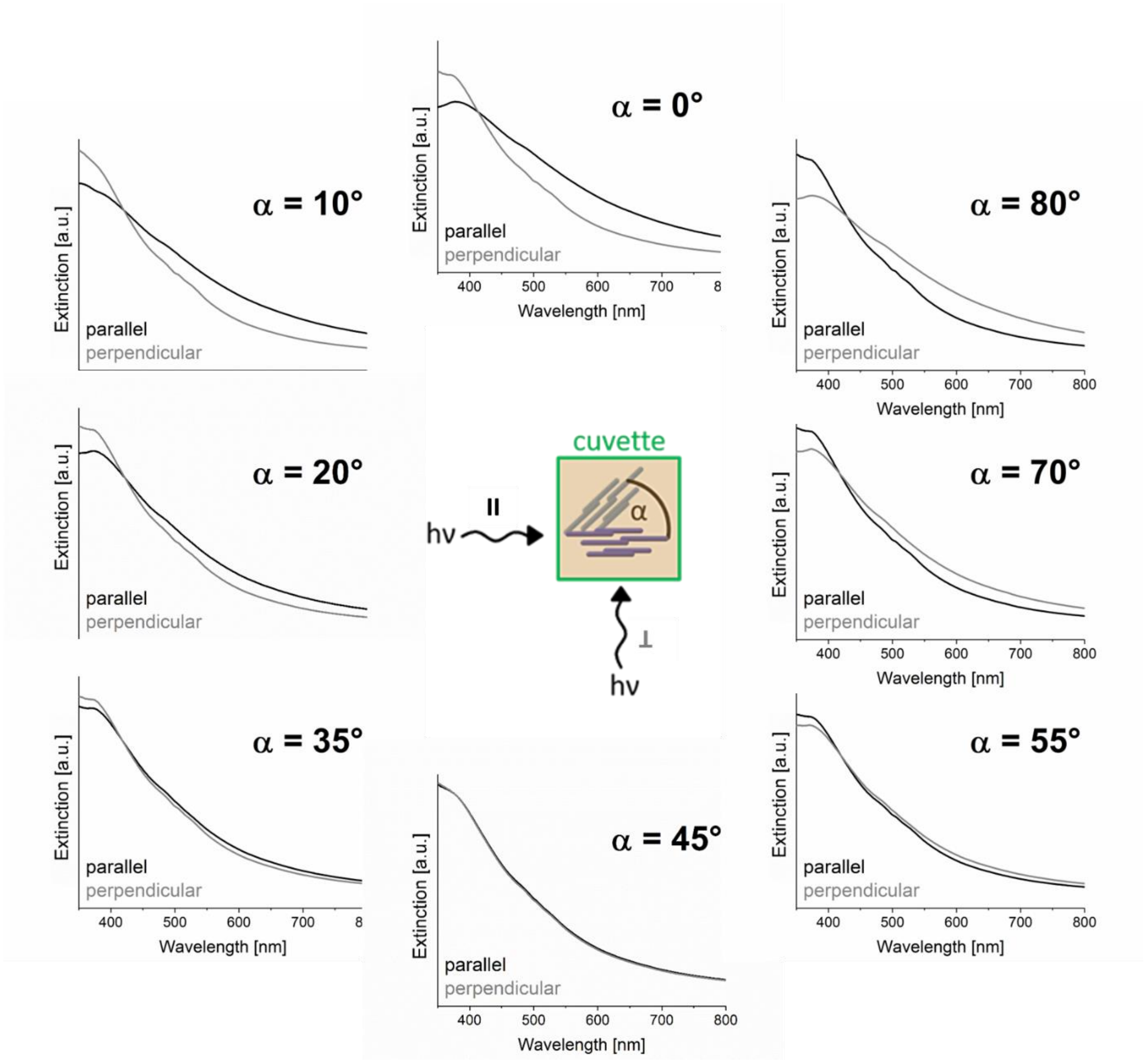

Figure S2. The width of splitting of the intensity of the curves below and above the wavelength of intersection varies depending on the orientation angle of the microrods to the incident light beams (which are oriented parallel II and perpendicularly ⊥ to the cuvette), however, the intersection is invariant to the change of illumination angle.

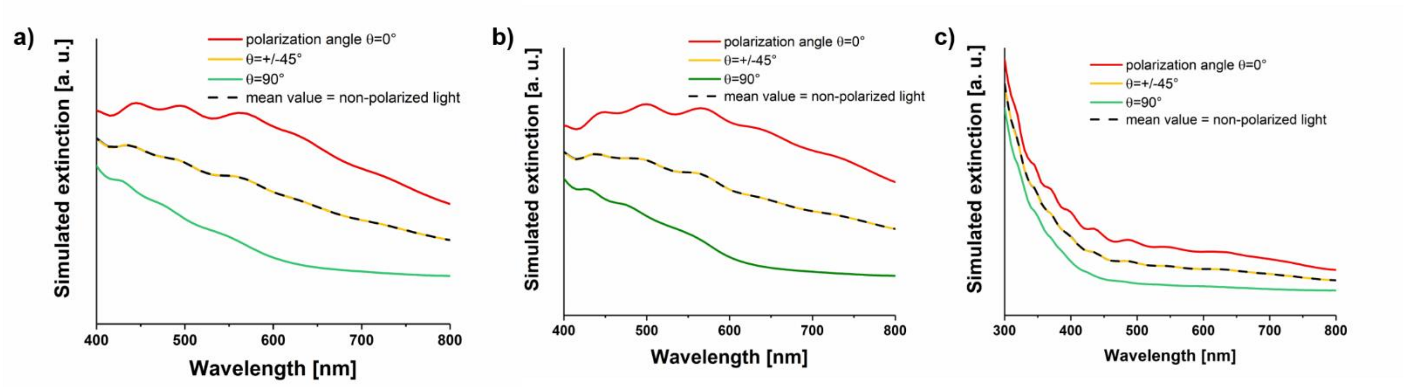

Figure S3. COMSOL simulated extinction cross-sections depending on the polarization direction -45°, 0°, 45° and 90° in the case of light propagation perpendicular to the microrod main axis and their mean value representing non-polarized light for Fe_2O_3 a) extraordinary and b) ordinary rays, as well as c) Fe_3O_4.

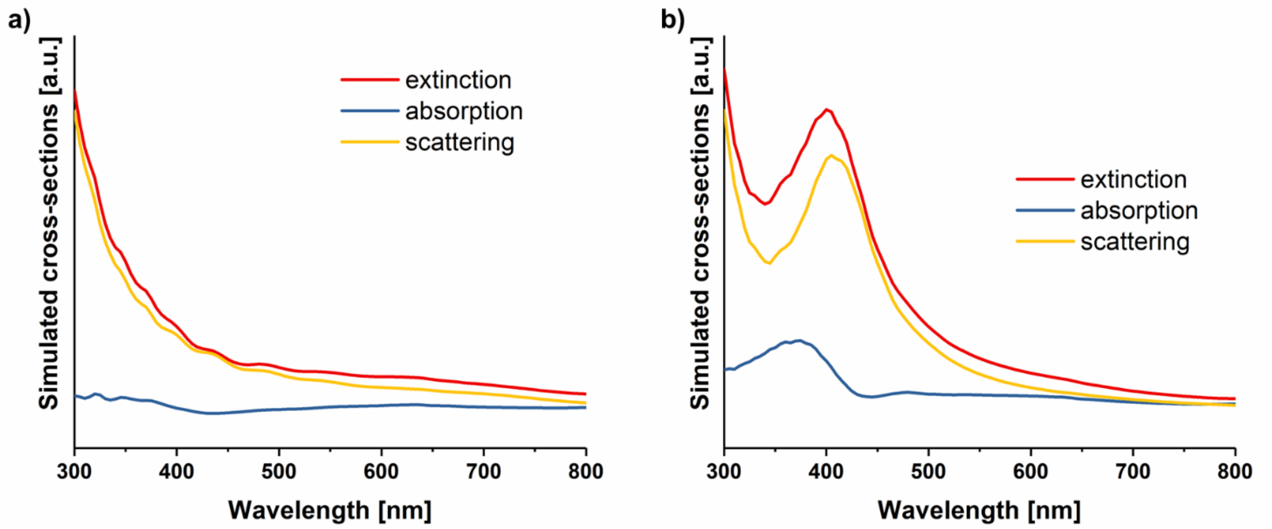

Figure S4. COMSOL simulated extinction, absorption and scattering cross-sections exemplarily shown for the case of light propagation a) perpendicular and b) parallel to the microrod main axis for a spheroid consisting of Fe_3O_4. The extinction cross-section is obtained by the addition of absorption and scattering.
